# Lipoma-Associated Intussusception of the Transverse Colon

**DOI:** 10.7759/cureus.38671

**Published:** 2023-05-07

**Authors:** Xiangliang Sui, Joan Q Tao, Jingjuan Min

**Affiliations:** 1 Biochemistry, Washington University in St. Louis, St. Louis, USA; 2 Radiology, University of Missouri School of Medicine, Columbia, USA; 3 Anesthesiology, St. Luke's Hospital, Chesterfield, USA

**Keywords:** computerized tomography, barium enema, abdominal pain, transverse colon, lipomatous lead point, intestinal intussusception, abdominal laparotomy

## Abstract

Intestinal intussusception rarely occurs in adults and is challenging to diagnose in the emergency department due to its associated nonspecific symptom of abdominal pain. Most of these incidences are caused by a neoplasm within the bowel acting as a lead point. Lipomas are benign fatty tumors that rarely develop in the colon and are very infrequently a precursor lesion to intussusception. Our present report describes a case of lipoma-associated intussusception in the transverse colon in an adult who presented with complaints of abdominal pain and acutely worsened chronic constipation. Computerized tomography (CT) imaging and barium enema revealed colocolonic intussusception with a lipomatous lead point and complete obstruction. The patient was admitted for same-day intervention and underwent a successful colectomy with no complications.

## Introduction

Intestinal intussusception is characterized by the invagination of a proximal bowel segment into the lumen of a distal bowel segment. Such a phenomenon was first documented by Dutch physician Paul Barbette in 1674 [1]. Intestinal intussusception more commonly affects pediatric patients than adult patients in a ratio of 20:1 [2]. These cases are typically managed through nonoperative reduction through air or contrast enemas. However, in very rare cases (2-3 cases/1,000,000 population/year), intestinal intussusception may occur in adults [3].

Clinical identification and diagnosis of intestinal intussusception is very challenging, given its presentation in the emergency department. Adult patients suffering from intestinal intussusception most commonly present with a nonspecific complaint of abdominal pain. High clinical suspicion, with corroboration through abdominal CT imaging, is needed to accurately diagnose this disorder.

In the majority of cases, the development of intussusception in adults is associated with a focal area of traction, such as a polyp or cancer, that draws the proximal bowel within the distal bowel during peristalsis [4]. This bowel ischemia leads to complications, including bowel necrosis and sepsis.

Intestinal intussusception most commonly affects the small bowel and rarely affects the large bowel. Sixty-six percent of focal areas for intestinal intussusception of the large bowel involve malignant neoplasms [4]. Benign tumors, such as lipomas, constitute a minority of cases. Lipomas are nonepithelial tumors composed of adipose cells which tend to favor fatty areas of the trunk, neck, and proximal extremities. Intestinal lipomas are rare and have a reported incidence of 0.2% to 4.4% [5].

We report on a case of an adult patient who presented to our emergency department with a chief complaint of intermittent, colicky abdominal pain over the past two weeks. His pain initially started in his lower left quadrant and then migrated to his right lower quadrant. A CT abdomen and pelvis with IV contrast showed a colocolonic intussusception between the ascending and transverse colon with a lipomatous lead point.

## Case presentation

The patient is a 70-year-old male with a medical history of benign prostatic hyperplasia and chronic constipation who presented to the emergency department (ED) with intermittent, colicky lower abdominal pain over the past two weeks. He explained that his pain started in his lower left quadrant before migrating to his right lower quadrant. The patient reported that he typically uses a suppository every three days for his chronic constipation, but it has acutely worsened such that he now needs to take one suppository daily. He endorsed ongoing abdominal pain while in the ED. He denied fever, chills, chest pain, shortness of breath, hematochezia, melena, diarrhea, or any history of diverticulitis. On ED arrival, the patient was hemodynamically stable (temperature: 36.4 C, heart rate (HR): 73, blood pressure (BP): 139/71, pulse: 73 bpm, respiratory rate (RR): 16, O2 saturation: 96% on room air). The patient's physical exam was positive for slight abdominal distention, serosanguinous JP drain, and bowel sounds.

**Figure 1:**
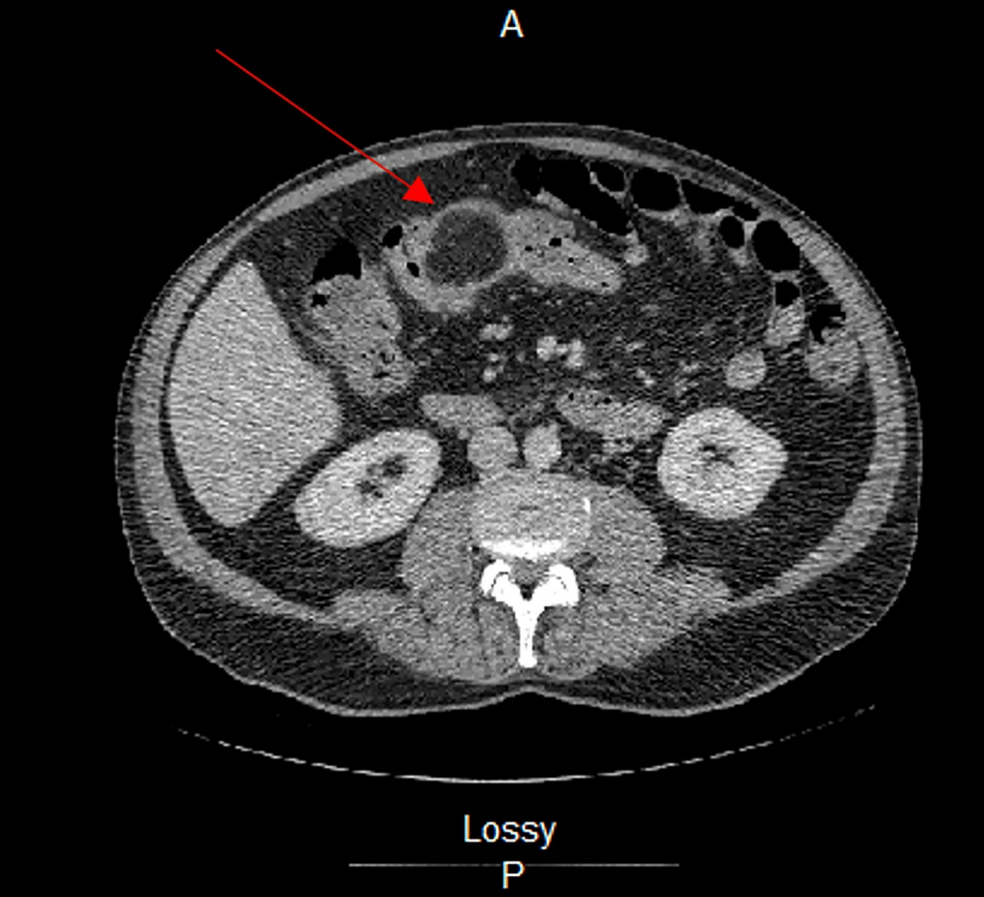
CT imaging with IV contrast revealed a benign lipoma located between the ascending and transverse colon. The lipoma presented as a well-circumscribed, thinly-encapsulated ovoid mass with low attenuation in the center.

**Figure 2:**
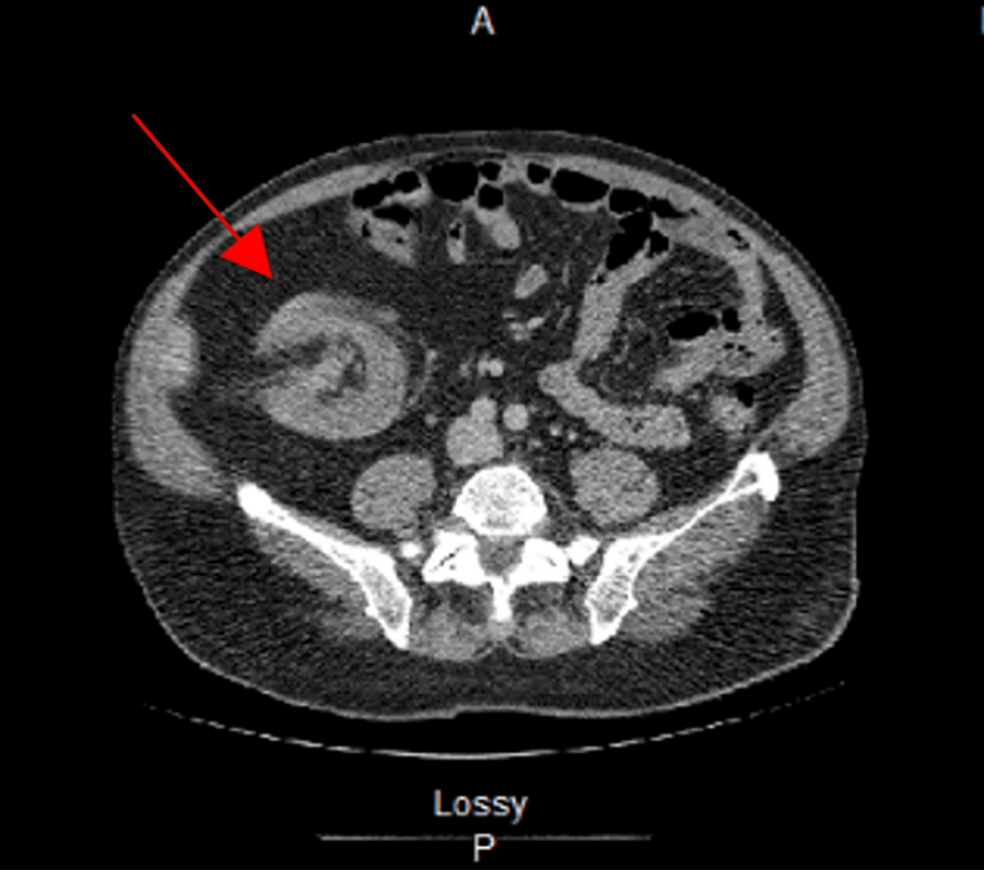
CT imaging with IV contrast demonstrated a proximal transverse colon obstruction resulting in a "bowel within a bowel" configuration which is consistent with intussusception.

**Figure 3:**
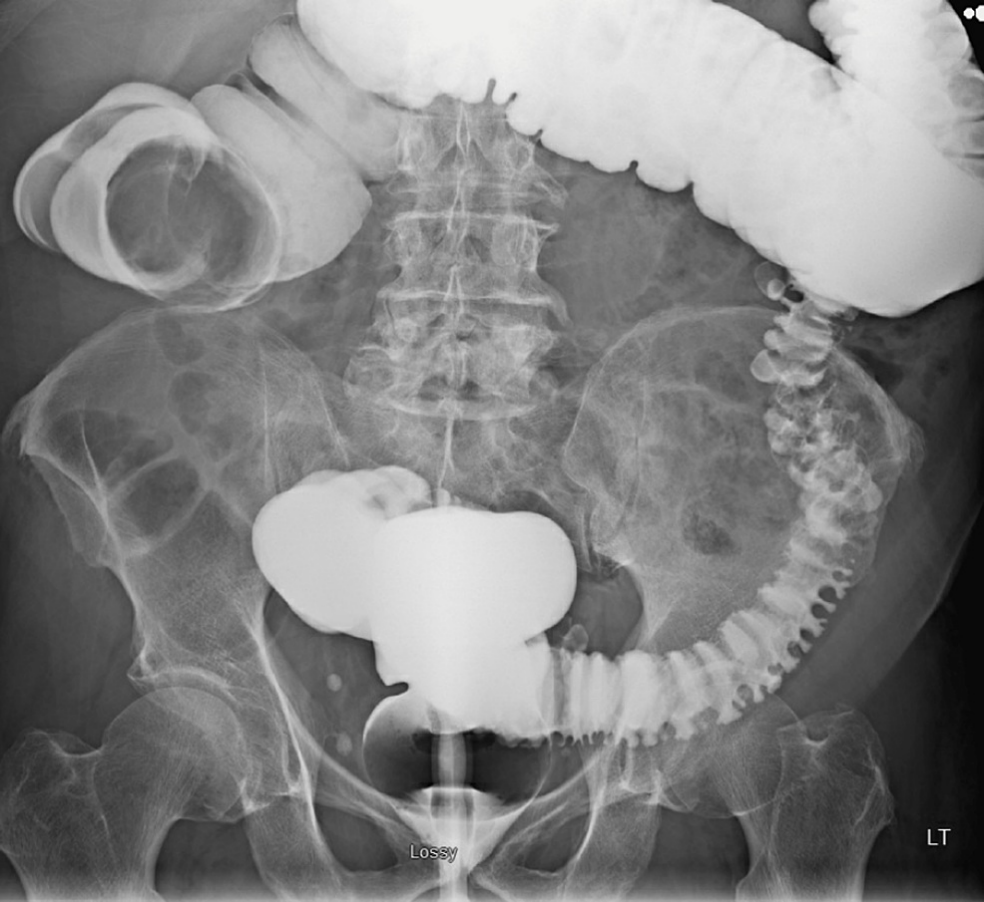
Barium enema followed by CT imaging revealed a round filling defect in the proximal transverse colon with complete obstruction. The location of the obstruction corresponded to a lipomatous lead point.

CT imaging of the abdomen and pelvis with IV contrast revealed a colocolonic intussusception between the ascending and transverse colon with a lipomatous lead point measuring 5.4 x 4.0 x 4.4 cm (Figures 1, 2). A barium enema was performed and demonstrated a round filling defect in the proximal transverse colon corresponding to the lipomatous lesion with complete obstruction (Figure 3).

**Figure 4:**
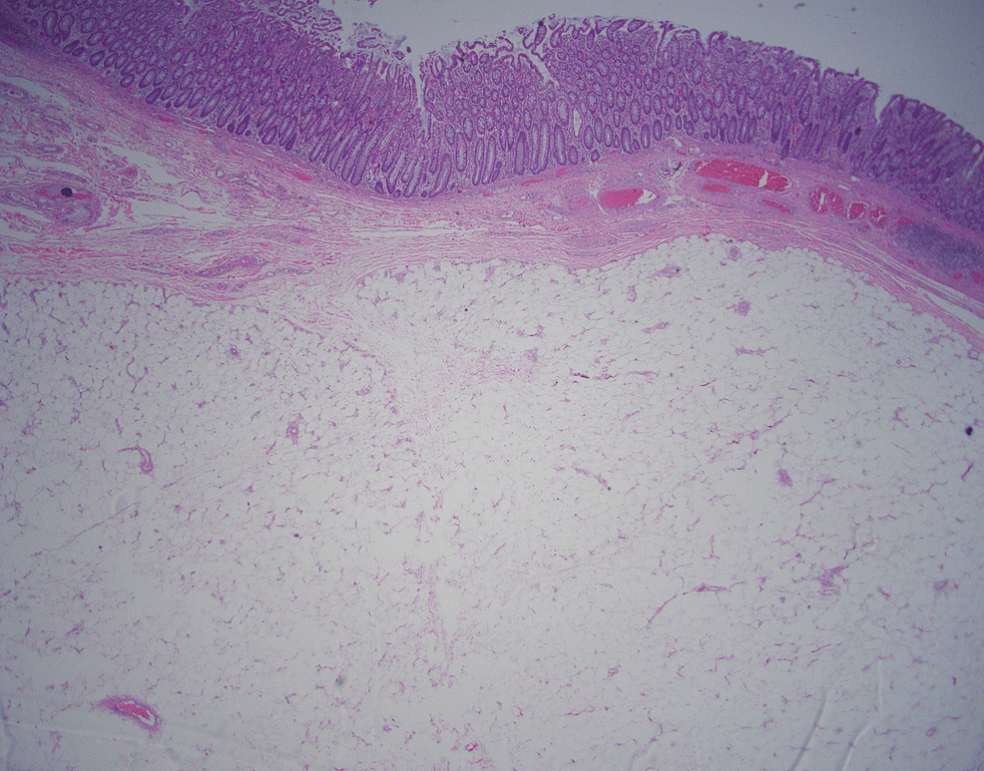
Histopathology of the resected specimen confirmed colonic epithelial cells with well-encapsulated adipose tissue that is consistent with a lipomatous mass.

The patient was admitted for same-day intervention and underwent a successful transverse colectomy with side-to-side functional end-to-end anastomosis. The middle transverse colon and the terminal ileum, approximately 20 cm from the ileocecal valve, was transected. Gross examination of the specimen showed a soft mass with well-defined margins. Histopathology confirmed well-encapsulated adipose tissue that is consistent with a lipoma (Figure 4). The patient had an uneventful postoperative course and was discharged home on the 10th day.

## Discussion

The presentation of intestinal intussusception in adults is unique in that it typically has a subacute or chronic onset of symptoms rather than acute onset, as seen in pediatric patients. Fewer than 20% of adults presenting with intussusception are found to have an acute complete bowel obstruction, and only 7-42% of cases exhibit a palpable abdominal mass [4,6,7]. Moreover, common complaints of intestinal intussusception are nonspecific, consisting of colicky abdominal pain, nausea, vomiting, and abdominal distention. As a result, diagnosis of this condition is challenging and requires a high degree of clinical suspicion. Exploratory laparotomy typically precedes diagnosis, and despite advances in medical imaging, only 32-50% of cases are diagnosed preoperatively [4,8-10]. While cases of pediatric intestinal intussusception are often primary or idiopathic, the majority of adult cases are secondary to structural lesions. Malignant neoplasms contribute to the majority of lead points of intussusception, causing 66% of colonic intussusceptions and 30% of cases in the small bowel [4]. Adenocarcinoma is the most common malignant lead point of colonic intussusception, whereas metastasis is the most common malignant lead point of small bowel intussusception. Other lead points of intussusception are rare but include benign tumors (lipomas, adenomatous polyps, fibromas, leiomyomas, hamartomas), post-surgical adhesions, lymphoid hyperplasia, cystic fibrosis, scleroderma, celiac disease, inflammatory bowel disease, appendicitis, pancreatitis, and rectal foreign bodies [8]. The intussusception of our patient was found to be secondary to a lipomatous lead point.

Lipomas are benign, nonepithelial tumors that are composed of adipose cells. These neoplasms present grossly as distinguished yellow masses. While lipomas may develop anywhere in the body, they tend to be found in the fatty regions of the trunk, neck, and proximal extremities [11]. The occurrence of lipomatous growth in the intestines is rare and has a reported incidence of 0.2% to 4.4% [5]. However, given that these benign neoplasms are typically asymptomatic and are often incidental findings, the actual incidence may be higher. Lipomas have a greater likelihood of becoming symptomatic when exceeding a diameter of 2 cm [12]. Moreover, 75% of intestinal lipomas that are greater than 4 cm in diameter are symptomatic. In these circumstances, lipomas may cause intestinal blockage, bleeding, or intussusception [3]. Prior studies have found that colonic lipomas most commonly occur in the ascending colon (45%). Lipomas occur less frequently in the sigmoid colon (30.3%) and descending colon (15.2%). Lipomas of the transverse colon, such as that of our patient, are the most uncommon (9.1%) [6,7,9,10,13,14].

Ultrasound is often the initial imaging modality for intussusceptions in both pediatric and adult populations. Ultrasound has advantages such as providing rapid, inexpensive imaging with real-time examination. However, ultrasound imaging depends on operator performance, and its efficacy may be reduced by bowel distention. Intussusception may appear on ultrasound imaging as a "target" or "donut" sign (transverse orientation), a "crescent in a donut" sign (longitudinal orientation), and a "pseudo-kidney" sign (oblique orientation) [15-17]. Although ultrasounds may alert early suspicion, preoperational diagnosis of intussusception is often confirmed with CT imaging. Presentation of intussusception on CT imaging will vary depending on the orientation. The most well-known sign is the "bowel-within-a-bowel" configuration, in which the layers of the bowel are duplicated, forming concentric rings when imaged at right angles to the lumen (equivalent to the ultrasound "donut" or "target" sign), and a soft tissue sausage when imaged longitudinally.

Pediatric intussusception management is often conservative; reduction is achieved through barium enemas rather than surgical intervention. In contrast, given the high incidence of lead point involvement and underlying malignancy, treatment of adult intussusception requires supportive measures (pain control, antiemetics, IV fluids, nasogastric (NG) tube) in addition to surgical intervention. The approach will vary depending on the location of the lesion:

Colocolic Intusussception

Treatment for colocolic intussusception is debatable. However, given the high incidence of underlying pathology, laparotomy rather than reduction is endorsed. There is still much controversy surrounding the reduction of intussusception lesions. Some studies favor an en-bloc resection over lesion reduction due to the risk of transperitoneal, vascular, and intraluminal seeding. Other studies believe that lesion reduction will reduce any unnecessarily extensive bowel resection [2,18].

Gastroduodenal Intusussception

Treatment for gastroduodenal intussusception requires the reduction followed by the surgical resection of the lead point [19].

Coloanal Intusussception

Physicians often agree on the reduction of the lesion first, followed by surgical resection. This approach is beneficial as it results in sphincter-sparing. For resection, an abdominal approach is most common; however, using a perianal and anal approach has increased in popularity [19].

Upon identification of the lesion, appropriate surgical intervention will typically resolve intestinal intussusception with minimal to no complications. However, idiopathic cases of adult intussusception without bowel obstruction may not require surgical intervention. In these patients, a conservative approach of admission with serial abdominal examinations may be appropriate to allow the intussusception to resolve without intervention [20].

## Conclusions

Intestinal intussusception is a condition that rarely occurs in adults and is difficult to diagnose in the ED. However, rapid identification of intussusception is necessary to avoid severe complications of bowel blockage, sepsis, or necrosis. Most adult intestinal intussusceptions occur in the small bowel and are associated with a malignant neoplastic lead point, and benign tumors, such as lipomas, are a very uncommon cause of intussusception. As such, our patient exhibited an exceptionally rare case of intestinal intussusception. Although the majority of intussusceptions are diagnosed through exploratory laparotomy, barium enema and CT imaging of our patient were sufficient to confirm intussusception of the transverse colon. Given that the intussusception was associated with a lipomatous lead point, surgical intervention was necessary. Our patient responded well to colonic resection and was ultimately discharged with no complications after a brief hospital stay.
